# Beyond a Zero-Sum Game: How Does the Impact of COVID-19 Vary by Gender?

**DOI:** 10.3389/fsoc.2021.650729

**Published:** 2021-06-15

**Authors:** Rosemary Morgan, Peter Baker, Derek M Griffith, Sabra L. Klein, Carmen H Logie, Amon Ashaba Mwiine, Ayden I Scheim, Janna R. Shapiro, Julia Smith, Clare Wenham, Alan White

**Affiliations:** ^1^ Department of International Health, Johns Hopkins Bloomberg School of Public Health, Baltimore, MD, United States; ^2^ Global Action on Men’s Health, London, United Kingdom; ^3^ Founder and Director of the Center for Research on Men’s Health and Professor of Medicine, Health and Society, Vanderbilt University, Nashville, TN, United States; ^4^ W. Harry Feinstone Department of Molecular Microbiology and Immunology and Department of International Health, Johns Hopkins Bloomberg School of Public Health, Baltimore, MD, United States; ^5^ Factor-Inwentash Faculty of Social Work, Canada and Women’s College Research Institute, Women’s College Hospital, University of Toronto, Toronto, ON, Canada; ^6^ School of Women and Gender Studies, Makerere University, Kampala, Uganda; ^7^ Department of Epidemiology and Biostatistics, Dornsife School of Public Health, Drexel University, Philadelphia, PA, United States; ^8^ Health Sciences, Simon Fraser University, Burnaby, BC, Canada; ^9^ Department of Health Policy, London School of Economics and Political Science, Leeds, United Kingdom; ^10^ Emeritus Professor of Men’s Health, Leeds Beckett University, Leeds, United Kingdom

**Keywords:** gender, sex differences, pandemic, COVID-19, LGBTQ

## Abstract

Epidemics and pandemics, like COVID-19, are not gender neutral. Much of the current work on gender, sex, and COVID-19, however, has seemed implicitly or explicitly to be attempting to demonstrate that either men or women have been hardest hit, treating differences between women and men as though it is not important to understand how each group is affected by the virus. This approach often leaves out the effect on gender and sexual minorities entirely. Believing that a more nuanced approach is needed now and for the future, we brought together a group of gender experts to answer the question: how are people of different genders impacted by COVID-19 and why? Individuals working in women’s, men’s, and LGBTQ health and wellbeing wrote sections to lay out the different ways that women, men, and gender and sexual minorities are affected by COVID-19. We demonstrate that there is not one group “most affected,” but that many groups are affected, and we need to move beyond a zero-sum game and engage in ways to mutually identify and support marginalized groups.

## Introduction

Epidemics and pandemics, like COVID-19, are not gender neutral (Wenham et al., 2020a). Much of the current work on gender, sex, and COVID-19, however, has seemed implicitly or explicitly to be attempting to demonstrate that either men or women have been hardest hit, while often leaving out the effect on gender and sexual minorities entirely (Bwire, 2020; Wenham et al., 2020b). Believing that a more nuanced approach is needed now, and will also be in the long term, we brought together a group of gender experts to answer the question: how are people of different genders impacted by COVID-19 and why? We also need to understand that sex and gender are different, and that when studying gender, it is necessary to move beyond binary approaches which place people into distinct categories. Each section below has been written by individuals working in the field of women’s, men’s, and LGBTQ health and wellbeing. We also asked experts on sex differences to contribute so that we could gain a better understanding of how sex intersects with gender to lead to greater understanding of gendered impact of COVID-19.

By laying out the different ways that women, men, and sexual and gender minorities are affected by COVID-19, we demonstrate that there is not one group “most affected,” but that many groups are affected depending on the context, and we need to move beyond a zero-sum game and engage in ways to mutually identify and support those effected. Most importantly, we need to use an intersectional approach (Bowleg, 2012; Bowleg, 2020; Griffith, 2012), which explores how gender intersects with other social stratifiers including race, age, income, disability, sexual orientation—to better understand and address individual and group experiences and effects of the pandemic.

## Understanding Sex and Gender

Biological sex is defined as “the classification of living things, generally as male or female according to their reproductive organs and functions assigned by chromosomal complement” (Springer et al., 2012b), while gender is the “socially constructed roles, behaviors, activities, attributes and opportunities that any society considers appropriate for men and women, boys and girls” and people with non-binary identities (WHO, 2020c). Gender is both rooted in biology and shaped by environment and experience (Springer et al., 2012b).

There are different approaches to how gender is framed, including categorical (e.g., binary traits or identities), relational (defined by relationships and interactions between and among men and women), and intersectional (a form of power relation which intersects with other social identities, such as race, age, and sexual orientation, to influence individual experiences of marginalization and disadvantage), or a combination thereof (Springer et al., 2012a). In earlier sociological accounts that sought to distinguish gender from sex, (West and Zimmerman, 1987) conceptualized gender as something “we do” rather than an essence that one naturally possesses. They argued: “Doing gender involves a complex of socially guided perceptual, interactional, and micro-political activities that cast particular pursuits as expressions of masculine and feminine (West and Zimmerman, 1987: 126). Heise et al. (2019) refer to gender as a social system, as opposed to a being considered a trait or identity. Gender systems define “men and women as different and distributes power, resources, and status on the basis of that difference” (Heise et al., 2019: 2,441). Patriarchal gendered systems typically distribute greater power, resources, and status to men and behaviors considered masculine. Those who do not fit into the recognized gender systems, such as gender minorities and/or men or women who do ascribe to traditional masculinities or femininities, are often granted less legitimacy and experience greater stigma and discrimination (Connell, 2005; Heise et al., 2019). In regards to the COVID-19 pandemic, gendered norms, roles, and behaviors that individuals conform to (or are expected to conform to) can influence risk of infection and exposure, as well as the social and economic impacts of pandemic response strategies.

## How are Women Impacted by COVID-19?

While context, cultural, socio-economic status and numerous identity factors differentiate women’s experiences of COVID-19, COVID-19 has also had particular effects on women that transcend borders and make explicit globalized structures of inequities. Around the world, women comprise 70% of the healthcare workforce and up to 90% of the social care workforce (WHO, 2020a). They are also more likely to be providing frontline care and are consequently at increased risk of COVID-19 infection–as is demonstrated by the analysis of cases among healthcare workers. For example, women account for 73% of COVID-19 infections among healthcare workers in Spain (UN Women, 2020). In India, women healthcare workers account for 38% of cases, even though they make up less than a third of the workforce (Dey and Pandit, 2020). At the Tonji Hospital in Wuhan, China, nurses (who are mostly women) had a 2.7-fold risk of contracting COVID-19 compared to physicians (who are mostly men) (Lai et al., 2020).

Globally women do two to three times more informal care work than men. In the context of COVID, they also absorbed expanding additional unpaid care work as a consequence of government responses to the pandemic (Lee and Frayn, 2008). COVID-19 related closures of schools in 193 countries, compounded by lockdown requirements, have increased the childcare and domestic work within households, and this falls disproportionately on women (Wenham et al., 2020b), due to the gender pay-gap, feminized sectors of the economy that were otherwise shut and social, cultural norms. Not only are women doing more of this domestic load, but research in the United Kingdom and Hong Kong has shown that women are suffering considerably worse mental health effects associated with this workload, particularly when juggling it alongside paid employment (Fawcett Society, 2020).

Care work in the home directly impacts women’s economic security, reducing their time and ability to engage in paid labor. A Canadian study found mothers were five times more likely than fathers to reduce work hours to care for children (Qian and Fuller, 2020). Similar trends have been demonstrated in Argentina, Europe and South Africa (Blaskó et al., 2020; Casale and Posel, 2020; Costoya et al., 2020). The long-term negative effects on women’s careers remain unknown, but evidence from North American demonstrates men are regain jobs lost due to COVID-19 at a much faster rate than women, particularly racialized women (Catalyst, 2020), and evidence from previous epidemics show that women remain out of the workforce for longer (Bandiera et al., 2019). Care work as a barrier to engaging in paid labor compounds the effects of a global economic downturn that is disproportionately affecting industries where the majority of workers are women, such as those of hospitality, restaurants, tourism and recreation (Franke, 2020).

A headline across the globe has been the soaring rates of calls to domestic violence hotlines during periods of lockdown (Chandra, 2020). Women are 90% of domestic violence victims and this extreme surge has been noted across continents. For example, in Colombia, calls to domestic violence hotlines have surged 130% (Reuters, 2020). Women are also disproportionately affected by COVID-19 through changes to access of sexual and reproductive health (SRH) services and maternal health care (Ahmed and Sonfield, 2020). The COVID-19 pandemic has affected both the supply and demand for SRH services. Supply chains for contraceptives, for example, have been disrupted, starting in Asia in early 2020, where most global contraceptives are produced. This resulted in stock-outs being reported, particularly in low and middle-income countries (LMICs) such as Myanmar and Mozambique (Purdy, 2020). This disruption is amplified by changes in demand, with quarantine orders and the closure of non-essential health services meaning that women may not be able to access contraceptives if they wish, or they may prefer not to seek these services, fearing clinics to be a location of transmission (Wenham et al., 2020b). In a context of scarce resources, quality and availability of health systems and fee for services costs will influence who has access to SRH and who does not. Further attention also needs to be paid to abortion access: some countries such as United States, Poland and Italy have used COVID-19 as an opportunity to further restrict women’s reproductive freedom, ruling abortion a non-essential service during the pandemic. On the other hand, in England the pandemic has liberalized abortion policy to facilitate medical abortions at home, thereby reducing demand on the health system (Margolis, 2020; Stevis-Gridneff et al., 2020).

Though there are stark global trends among women in different contexts, experiences will be further structured by location (both in terms of national, urban or rural and neighborhood context), socio-economic status, race, citizen status, age, sexual orientation and gender identity, ability and other intersecting factors. In the United Kingdom, data from England and Wales shows Black women are 4.3 times more likely to die from a COVID-19-related death than white women (Office for National Statistics, 2020). Similar data from the US shows disproportionate rates of COVID-19 related deaths among black women, compared to white men (Rushovich et al., 2021). In Hong Kong, foreign domestic workers were ineligible to receive government COVID-19 support, even as large numbers lost their jobs when their employers left the city (Milhaud, 2020). Sex workers, the majority of whom are women, are also excluded from most government support programs due to the criminalization of their profession. Girls in LMICs are particularly put at risk by school closures, with (Save the Children, 2020) predicting that up to 2.5 million more girls around the world are at risk of being forced into child marriage over the next five years. Lesbian women, particularly in places where same sex behaviors are criminalized and lead to discrimination, may be particularly affected by isolation policies that separate them from partners and chosen families. The list of differential impacts among women could go on and requires nuanced detailed and dedicated analysis of the intersecting factors that structure women’s risks. While global trends paint a clear outline of how COVID-19 is exacerbating the inequalities women around the world face, further analysis is needed to fill in the details.

## How are Men Impacted by COVID-19?

Men are significantly more likely than women to develop severe outcomes associated with COVID-19 and to die, with globally 13 male deaths for every 10 female deaths and 18 male ICU admissions for every 10 female ICU admissions (Global Health 50/50, 2020; WHO, 2020b). There has been considerable discussion about the biopsychosocial factors responsible for the disproportionate male mortality rate from the pandemic (Griffith et al., 2020; White and Kirby, 2020). An important component of men’s vulnerability to COVID-19 is the effect of biological sex, as male susceptibility to COVID-19 is likely mediated by the genetic and hormonal influences, which are discussed in greater detail below.

These biological effects, however, are often influenced by masculinities and gendered practices, norms and policies, and how race, ethnicity, socioeconomic position, and other factors intersect with gendered structures (Griffith, 2016; Griffith, 2020; Griffith et al., 2020; Smith et al., 2020). Consequently, men who are most socially and economically disadvantaged, have much higher mortality rates, as do men from Black and ethnic minority communities (Public Health England, 2020). While it is common to consider these biological and socio-cultural factors separately, patterns of COVID-19 mortality have illustrated the importance of using an intersectional lens that considers how race/ethnicity, sexual orientation, employment sector, place/country and other factors intersect with sex and gender to identify men at risk more precisely (Bajunirwe et al., 2020; Goldblatt and Morrison, 2020; Islam et al., 2020).

The greater risk of the severe form of the disease and the higher death rates in men is not the whole story (White, 2020). The pandemic’s impact on “normal life” and the looming potential economic recession is having a significant impact on men’s mental health. One international study found that the three most common causes of anxiety for men were the health of vulnerable relatives, falling ill, and losing their job (Movember, 2020). Alcohol-specific deaths in men have risen significantly during lockdown in England and Wales reflecting increases in consumption, particularly among already heavy drinkers (Breen and Manders, 2021). There is a concern that the economic recession caused by the pandemic will, as with previous recessions (Reeves et al., 2015), result in a marked increase in suicide rates among men (Khan et al., 2020). Lockdowns have already been linked to an increase in violence perpetrated by men against women and girls (UN Women, 2020). While the impact on women and girls is rightly a priority issue to address, such violence is also associated with increased stress and mental illness among men (Peterman et al., 2020). In Eastern African countries, Uganda in particular, a study on men and GBV during the pandemic noted unreported violence cases experienced by men as well as social and economic stress and anxiety triggered by the unchanging expectations of men as providers amid pandemic job losses and restricted mobility (Ahikire and Mwiine, 2020). The emphasis of policy and programmatic initiatives has been on criminalizing men rather than developing and disseminating successful prevention and intervention programmes (Heilman and Barker, 2020).

The pandemic is revealing the woeful lack of attention paid to promoting men’s health; for example, little in the way of planning outreach to men with gender and broader intersectionality-sensitive health promotion advice, or focus on employment in risky male-dominated settings such as cross-border truck driving (Abalo, 2020), security work, meat processing plants, bus and taxi-driving (Burdorf et al., 2021). Interventions for men could include “male-friendly” messaging on handwashing, social distancing, wearing facemasks, accessing testing for COVID-19 infection, encouraging appropriate use of health services, as well as the mitigation of occupational risks (Griffith, 2020). However, although such broad-brush approaches can get messages out to the male population in general, for success there has to be a more nuanced targeting of messaging that recognizes how men of different ages, ethnicities, sexuality, disability and other lived experiences will respond. In addition, attention is needed to address the lag in COVID-19 vaccination among men in countries like the United States (Law, 2021).

The WHO has called for gender-responsive actions (WHO, 2020b) but, as yet, there is no evidence that these have been forthcoming. While COVID-19 provides further evidence of the need for gender mainstreaming in health policy (Varanka, 2008; White and Richardson, 2011), it also has highlighted the need for policy and planning to explicitly consider the diverse needs and interests of different categories of men (Smith et al., 2020). In the longer-term, health systems must develop a systematic approach to sex and gender that includes taking appropriate account of men’s health needs alongside those of women and non-binary genders. Policies and practices are required to tackle the deep-seated causes of poor male health and premature mortality. This includes developing a better understanding of, and then tackling, the structural causes that put men at additional risk of death from COVID-19 and its wider repercussions.

## How are Gender and Sexual Minorities Impacted by COVID-19?

Much of the attention to sex and gender in relation to COVID-19 has been related to the impact on (presumed cisgender, heterosexual) women and men, while often leaving out the effect on gender and sexual minorities entirely (Cahill et al., 2020). But gender affects everyone. Here we have included both gender and sexual minorities due to the intertwined nature of gender and sexual orientation, which can sometimes be difficult to disentangle. The two are frequently conflated leading to similar patterns of marginalization and discrimination among this population. Intersectional approaches also signal the need to consider the intersection of sexual orientation and gender alongside other social identities and categories including race, socio-economic status, and migration status.

To understand the impact of COVID-19 on gender and sexual minorities a more nuanced and intersectional approach is needed. Lesbian, gay, bisexual, and transgender (LGBT) persons experience global health inequities rooted in structural stigma targeting LGBT identities alongside other axes of marginalization such as racism, sexism, and classism. The social, legal, and healthcare environment for LGBT persons varies widely within and between countries; however, globally discrimination and exclusion converge to worsen health while reducing access to care (Lucas Ramón Mendos and ILGA World, 2019). These pre-existing social and health disparities indicate that COVID-19 may disproportionately impact LGBT persons. We briefly identify challenges experienced by LGBT persons in the context of COVID-19.

First, LGBT persons may experience elevated risks for poor COVID-19 outcomes. For instance, LGBT persons are more likely to smoke compared with heterosexual, cisgender persons (Broverman, 2020; Whittington et al., 2020), and may have elevated cardiovascular disease risk (Caceres et al., 2017; Meads et al., 2018). Gay and bisexual men, and transgender (trans) women are overrepresented among persons living with HIV (UNAIDS, 2020b) who may experience respiratory and cardiovascular multi-morbidities (Cahill, 2020). Importantly, Black, Indigenous, and other racialized gay and bisexual men and transgender women are most impacted by HIV in many countries and it is those same racialized groups that are disproportionately burdened by COVID-19 (Bowleg, 2020; Islam et al., 2020). Further, COVID-19 threatens access to HIV prevention, testing and care services (UNAIDS, 2020a). A large online study of men who have sex with men (MSM) (*n* = 2,732) from 103 countries reported that 33% of participants living with HIV lost access to HIV providers due to COVID-19, and nearly one-fifth who were taking antiretroviral therapy reported challenges accessing medication (Santos et al., 2020). In another survey of MSM (*n* = 10,654) in 20 countries using geosocial networking apps, interruptions to HIV prevention services were common (e.g., 56% for pre-exposure prophylaxis, 38–55% for HIV testing) and significantly associated with the stringency of national restrictions related to COVID-19 (Rao et al., 2021). LGBT persons may also avoid COVID-19 testing or emergency care due to pre-existing fears of mistreatment in health facilities (Bauer et al., 2014). In a Canadian survey of 820 transgender and non-binary persons, 10.8% reported experiencing discrimination when accessing or attempting to access COVID-19 testing (Trans PULSE Canada, 2020).

Mental health effects are another concern. Quarantines and closures of LGBT spaces may elevate depression and anxiety while reducing access to social support (Brennan et al., 2020). In a US study of MSM, 69% reported decreased quality of life, 73% reported increased anxiety, and 56% reported feeling less connected to friends since COVID-19 (Sanchez et al., 2020). In a longitudinal cohort of LGBT people in the US, among people who did not have pre-existing depression or anxiety disorders, depression and anxiety symptoms increased during to the pandemic (Flentje et al., 2020). Globally, gender affirming surgeries have been cancelled or postponed for many trans persons (Streed and Siegel, 2020), which can elevate anxiety and depression (Wang et al., 2020). Indeed, in an online global sample of 849 trans and non-binary individuals, reduced access to gender-affirming medical care and supplies, as well as socioeconomic loss, due to the pandemic were associated with poorer mental health (Restar et al., 2021). LGBT individuals, particularly youth, who are isolating with unsupportive family members may be at risk of experiencing violence or distress (Action International, 2020; Ahlenback, 2020; OutRight). Researchers have called for further investigation of how stay-at-home orders impact mental health and experiences of violence among sexual and gender minorities, particularly among adolescents who may have limited access to supportive peer groups (DeMulder et al., 2020). Santos et al. (2020) global MSM survey reported that more than one-third (35%) reported depression symptoms, and this rose to 50% among those who lost their employment due to COVID-19.

Socio-economic marginalization of LGBT persons is likely to be exacerbated by the pandemic. For instance, Stonewall (2020) report noted lower educational attainment, poverty, and housing and food insecurity among lesbian and bisexual women and trans persons related to stigma and discrimination across 26 countries. LGBT persons are overrepresented within sectors considered nonessential (e.g., food service) (Cahill, 2020; Whittington et al., 2020), and within occupations curtailed or made riskier by physical distancing protocols (e.g., sex work) (Goel, 2020). Finally, legal and policy responses to COVID-19 may compound impacts of the pandemic on LGBT persons. For instance, Panama and Peru enacted mobility policies whereby men and women were permitted to leave their homes on separate days; these laws resulted in police brutality and public humiliation targeting trans and gender nonconforming persons (Perez-Brumer and Silva-Santisteban, 2020). Abuse and punishment by officials enforcing quarantine and curfews disproportionately targeted and punished LGBT persons in Panama (Reid, 2020), Philippines (Reid, 2020), and Uganda (The Lancet, 2020). LGBT community members became scapegoats for COVID-19 transmission in a number of countries (BRAC, 2020; Goel, 2020; The Lancet, 2020).

Together these data signal that LGBT persons are in many ways disproportionately impacted by COVID-19 and its socio-economic consequences. The largest empirical COVID-19 studies with LGBT persons, however, have focused on cisgender men (Sanchez et al., 2020; Santos et al., 2020). Amplifying voices and experiences of COVID-19 among trans persons, and lesbian and bisexual women, and intersections with race, Indigeneity, ability, and other axes along which COVID-19 vulnerability is unequally distributed, is urgently needed to inform practice and policy to achieve health equity.

## The Role of Biological Sex

To fully understand the impact of COVID-19 on women and men, one must also consider the role of biological sex. This is because sex and gender are so inherently intertwined, each affecting the other in complex ways. Most of the health effects we see are not the result of sex or gender but the entanglement or intersection of the two (Springer et al., 2012). As our understanding of biological susceptibility beyond the sex/gender binary is limited, we do not consider the role of biological sex on gender minorities.

While gender places women at a disadvantage during the pandemic in many ways, female sex appears to offer some protection against severe disease and death from COVID-19. This biological sex difference is likely mediated by both genes and hormones. Viral infections begin with the virus binding to a receptor that is expressed on human cells. For SARS-CoV-2, the receptor is angiotensin-converting enzyme 2 (ACE2), a protein that is down-regulated by estrogens and expressed from a gene coded on the X-chromosome (Liu et al., 2010). As with all X-linked genes, males inherit a single version from their mothers, while females inherit a version from each parent. To accommodate the extra copy in females, each cell inactivates one X-chromosome creating a mosaic of expression whereby some cells express the maternal copy and others express the paternal copy (Gibson et al., 2020). Therefore, if a variant of ACE2 that is better able to bind SARS-CoV-2 is inherited, males will express it in all cells, while females will only express it in half of their cells, leading to greater vulnerability in males (Gibson et al., 2020; Li et al., 2020). In addition, in females, genes can escape X-inactivation, leading to increased expression of X-linked genes. In addition to ACE2, toll-like receptor 7 (TLR7), is encoded on the X-chromosome. Both ACE2 and TLR7 have been shown to escape X-inactivation in immune cells, allowing females to express both copies and conferring a greater ability to sense intracellular viruses (Souyris et al., 2018; Li et al., 2020). The virus’s attachment onto ACE2 is the key route into the cell and its interaction with the enzyme also damages its normal protective function. With females potentially having two forms of the ACE2 enzyme (one from each X-chromosome), they have a greater likelihood of having unaffected ACE2 circulating, reducing the virus’s damaging effects (Li et al., 2020).

Sex differences also continue as disease progresses. In several retrospective cohort studies in China, the virus persisted longer in males than in females with severe disease (Shi et al., 2020; Xu et al., 2020; Zheng et al., 2020). This is because, while the immune response is critical to containing infection, it can also become dysregulated and contribute to disease progression. There is accumulating evidence to suggest that severe disease and death from COVID-19 are mediated by an excessive inflammatory response, termed a “cytokine storm” (Ye et al., 2020). Cytokine storms are characterized by rapid infiltration of immune cells in the lungs, leading to acute respiratory distress syndrome (ARDS) and ultimately multi-organ failure (Ye et al., 2020). Hormone-mediated sex differences may exist in the development of cytokine storms. In both humans and animal models, estrogens, including estradiol, have anti-inflammatory effects that inhibit the pro-inflammatory response [reviewed in Mauvais-Jarvis et al., (2020)], potentially protecting females from severe disease. This does not, however, explain female protection at later stages of life (e.g., during the post-menopausal period) when estrogen levels are low.

Table 1 provides a summary of the gendered impacts on women, men, and gender and sexual minorities discussed above.

**TABLE 1:** Summary of gendered impacts on women, men, and gender and sexual minorities.

Women	Men	Gender and sexual minorities
Increased rates of infection	Increased disease severity and mortality	Risk of poor outcomes
Women comprise majority of health and social care workforce and provide more frontline care leading to increased risk of infection.	Men are significantly more likely than women to experience severe disease and to die from COVID-19. This disparity is mediated by genetic and hormonal influences. Men’s susceptibility to certain underlying conditions (e.g., hypertension) is also a factor.	LGBT persons may experience elevated risks for poor COVID-19 outcomes to inequitable social contexts and healthcare discrimination that contribute to stress and pre-COVID-19 health disparities; for instance, LGBT persons are more likely to smoke compared with heterosexual, cisgender persons, and may have elevated cardiovascular disease risk.
Increased informal care	Increased vulnerability and risk	Increased vulnerability and risk
Women engage in more informal care work than men and faced additional unpaid care work during pandemic, including childcare and domestic work.	Men who are most socially and economically disadvantaged and those from black and ethnic minority communities have much higher mortality rates. Men in certain occupations (eg. transport) are also more at risk.	Black, indigenous, and other racialized gay and bisexual men and transgender women may be disproportionately burdened by COVID-19 due to existing social and health disparities and intersecting stigma and discrimination across social/health spheres.
Economic insecurity	Harmful masculinities and gendered practices, norms and policies also can leave men more vulnerable.
Women’s additional care work increases economic insecurity due to decreased opportunities for paid labor.	Mental health burden	Access to services impacted, including:
The majority of workers employed in industries which shut down during pandemic were women.	Men’s mental health impacted by cessation of normal life and the looming potential economic recession. Concern that will result in increase in suicide rates among men. There is evidence of increased alcohol-related diseases in men during pandemic.	HIV prevention, testing and care services which can harm HIV clinical health outcomes, and gender affirming surgeries, which can elevate anxiety and depression.
Violence	Violence	Mental health burden
Women experienced increased rates of domestic and healthcare worker violence.	Lockdowns have been linked to an increase in violence perpetrated by men against women and girls, which is associated with increased stress and mental illness among men.	Increased mental health burden and reduced access to social support due to closures of LGBT spaces.
Access to sexual and reproductive health services	Economic insecurity	Violence
The pandemic affected access to sexual and reproductive health services, including access to contraception and abortion services; while sexual and reproductive health affects all genders, women usually bear the responsibility for accessing contraception and health services, and most severe consequences of lack of access.	Unchanging expectations of men as providers amid pandemic job losses and restricted mobility triggered tensions in majority of households in LMICs.	LGBT individuals, particularly youth, who are isolating with unsupportive family members may be at risk of experiencing violence or distress.
This also extends to access to maternity services, with service provision halted in many locations with impacts on maternal and neonatal outcomes.	Gender imbalance in vaccination	Economic insecurity
Early marriage	Many men are less likely that women to race for covid-19 vaccination, with low rates influenced, in part, by men’s past experiences of healthcare seeking.	LGBT persons are overrepresented within sectors considered nonessential (e.g., food service) and within occupations curtailed or made riskier by physical distancing protocols (e.g., sex work).
Girls in LMICs at increased risk of forced marriages especially with closure of schools.	Absence from policy agendas	Stigma and discrimination
Increased vulnerability and risk	Men tend to be invisible in policy making, with little attention paid to how to reach out and target them more effectively.	Avoidance of COVID-19 testing or emergency care due to anticipated stigma and mistreatment in health facilities.
Minority women, including black women, lesbian women, foreign domestic workers, sex workers, have been disproportionately impacted by the above.		Legal and policy responses to COVID-19 may compound impacts of the pandemic on LGBT persons, such as mobility policies which permit men and women to leave homes on separate days.

## Taking a Holistic Response to Sex and Gender

What is clear is that people are disproportionately impacted in different ways—by infection and mortality from the pathogen and from longer term socio-economic effects. We need to recognize that these impacts, while different, do not necessarily equate to one group of people being “more impacted” than the other. The situation is much more complex than that, yet what remains clear is that gender does influence different primary short-term and secondary long-term effects of the pandemic. Primary effects include greater severity of disease and mortality among men, while secondary effects include higher social and economic consequences for women. And there is urgent need for more data on primary and secondary effects on gender and sexual minorities. When discussing the gendered impacts of pandemics, we must not only look at differences between but also among men, women and gender non-binary persons by considering how gender intersects with other biological and social stratifiers–like sex, race, age, gender identity, income, disability, sexual orientation—to create individual experiences of marginalization and vulnerability (Smith et al., 2020). The degree to which populations are affected will depend on their circumstances, which are shaped by wider historical and contemporary systems and structures of oppression and privilege.

COVID-19 has brought increasing attention to the role of sex and gender in health and the need for sex and gender disaggregated data and analysis (Gebhard et al., 2020; Griffith et al., 2020). Over the past year, papers and news articles have drawn attention to the role of sex and gender in relation to COVID-19. This is despite the fact that feminist scholars and advocates in the fields of men’s and women’s health have been arguing for the need for sex and gender disaggregated data and analysis for years, and it being agreed upon at the World Health Assembly Resolution 60.25 (2007). Why has it taken COVID-19 to finally show the importance of sex and gender-based analyses?

There are a number of possible reasons for this. One is likely due to the issue of power and who has historically set the agenda. Those who hold social identities which are given more power and privilege (i.e., white, male, high income, etc.) are overrepresented in decision-making (Bali et al., 2020). One can argue that those in positions of power typically prioritize issues that are in their interest. Sex and gender are often incorrectly equated with women and girls, despite the fact that sex and gender differences also affect men and gender non-binary persons, and that there is sexual diversity across the gender spectrum (Baker et al., 2020). Sex and gender have simply not been prioritized. In addition, for years data has been analyzed using the default (white) male as a reference point. Not only were women often left out of clinical trials, but studies using mice typically only used male mice (Klein and Morgan, 2020). Recommended dosages and signs and symptoms of ill health which were considered universal were in fact based on the male body. This has had negative consequences for women, not to mention for gender and sexual minorities. But men’s health has also remained an overlooked issue with just four countries having national men’s health policies and few addressing key issues such as men’s excess all-cause premature mortality burden (Baker et al., 2020).

This also draws attention to the complex issue of when to take a binary perspective and when we must move beyond the binary (Santos, 2014; Liszewski et al., 2018; Hart et al., 2019; Scandurra et al., 2019). For studies involving biological sex, while we recognize a more binary approach may be important due to the distinct ways that genes and hormones affect female and, males differently, a non-binary approach should be taken whenever possible so as to not exclude intersex people (Jorge et al., 2019; Costello, 2020; Joel, 2021). Due to the socially constructed and context specific nature of gender, and the ways in which it intersects with other social stratifiers, for studies involving gender taking a binary approach is no longer adequate and is in fact bad science. We must continue to fight for this distinction as sex and gender continue to be conflated.

Table 2 outlines key recommendations for addressing gendered impacts of COVID-19 based on the discussion above.

**TABLE 2:** Recommendations for addressing gendered impacts of COVID-19.

Recommendations	
Cross-cutting	Gender mainstreaming in health policy, including policy and planning explicitly considering the diverse needs and interests of different categories of men, women, and sexual and gender minorities.
	Collection and analysis of sex and gender disaggregated data.
	Data collection and analysis which include gender and feminist methodologies to capture the lived realities of communities and individuals otherwise missed.
	Biomedical research to understand underlying mechanisms of sex differences in disease severity and mortalty.
	Intersectional analysis which looks at differences among different categories of men, women, and sexual and gender minorities.
	Nuanced targeting of messaging that recognizes how men of different ages, ethnicities, sexuality and disability etc. will respond.
	Include diverse representation in decision-making.
	Support for non-governmental organizations specializing in reaching specific gender groups.
Women	Increased attention to the socio-economic effects of government interventions and recognition of the impacts on women.
	Social support mechanisms established to minimize economic harms to women unable to work, and future planning for how to ensure the longevity of employers/sectors which disproportionately employ women (e.g., sector wide bailouts) or training schemes for women.
	Minimum service package of sexual and reproductive health and maternity services to continue during health emergencies.
	Additional service provision for domestic violence support and protection
	Care based economic development to recognize the formal and informal care work that women perform upon which our society depends.
Men	Increased attention to the health of men and boys needed, including increased planning outreach to men with gender and broader intersectionality sensitive health promotion advice.
	Focused health promotion in male-dominated employment settings such as cross-border truck driving, meat processing plants, and bus and taxi-driving.
	Interventions for men which include “male-friendly” messaging on handwashing, social distancing, wearing facemasks, accessing testing for COVID-19 infection, encouraging appropriate use of health services, as well as the mitigation of occupational risks.
	Policies and practices are required to tackle the deep-seated causes of poor male health and premature mortality, including developing a better understanding of, and then tackling, the structural causes that put men at additional risk of death from COVID-19.
Sexual and gender minorities	Urgent need for more data on primary and secondary effects on gender and sexual minorities, with a focus on adolescents, and the different experiences among lesbian, bisexual and queer women, gay and bisexual men by gender identity, race, socio-economic status, regionality.
	Focus in women’s health to consider transgender women and lesbian, bisexual and queer women, and in men’s health to consider sexual minority men and transgender men.
	Need to move beyond binary sex and gender to be inclusive of gender non-binary persons and intersex persons, and to understand the experiences of gender non-binary and intersex persons in COVID-19.

One has to wonder whether the fact that COVID-19 is causing wide ranging effects of sex and gender will result in leading to greater attention to sex and gender. Will decision-makers, researchers, and practitioners finally begin to prioritize the role sex and gender play in the health of all? While we have yet to see this, we hope that the increased attention will lead to a more equitable pandemic response.
